# Repetitive nociceptive stimulation increases spontaneous neural activation similar to nociception-induced activity in mouse insular cortex

**DOI:** 10.1038/s41598-022-19562-1

**Published:** 2022-09-07

**Authors:** Shutaro Kobayashi, Kazunori O’Hashi, Masayuki Kobayashi

**Affiliations:** 1 Department of Pharmacology, Nihon University School of Dentistry, 1-8-13 Kanda-Surugadai, Chiyoda-ku, Tokyo, 101-8310 Japan; 2 Department of Oral Surgery, Nihon University School of Dentistry, 1-8-13 Kanda-Surugadai, Chiyoda-ku, Tokyo, 101-8310 Japan; 3 Division of Oral and Craniomaxillofacial Research, Dental Research Center, Nihon University School of Dentistry, 1-8-13 Kanda-Surugadai, Chiyoda-ku, Tokyo, 101-8310 Japan; 4 Department of Mental Disorder Research, National Institute of Neuroscience, National Center of Neurology and Psychiatry (NCNP), 4-1-1 Ogawa-Higashi, Kodaira, Tokyo, 187-8502 Japan; 5 Molecular Imaging Research Center, RIKEN, 6-7-3 Minatojima-minamimachi, Chuo-ku, Kobe, 650-0047 Japan

**Keywords:** Neuroscience, Sensory processing

## Abstract

Recent noninvasive neuroimaging technology has revealed that spatiotemporal patterns of cortical spontaneous activity observed in chronic pain patients are different from those in healthy subjects, suggesting that the spontaneous cortical activity plays a key role in the induction and/or maintenance of chronic pain. However, the mechanisms of the spontaneously emerging activities supposed to be induced by nociceptive inputs remain to be established. In the present study, we investigated spontaneous cortical activities in sessions before and after electrical stimulation of the periodontal ligament (PDL) by applying wide-field and two-photon calcium imaging to anesthetized GCaMP6s transgenic mice. First, we identified the sequential cortical activation patterns from the primary somatosensory and secondary somatosensory cortices to the insular cortex (IC) by PDL stimulation. We, then found that spontaneous IC activities that exhibited a similar spatiotemporal cortical pattern to evoked activities by PDL stimulation increased in the session after repetitive PDL stimulation. At the single-cell level, repetitive PDL stimulation augmented the synchronous neuronal activity. These results suggest that cortical plasticity induced by the repetitive stimulation leads to the frequent PDL stimulation-evoked-like spontaneous IC activation. This nociception-induced spontaneous activity in IC may be a part of mechanisms that induces chronic pain.

## Introduction

Recent neuroimaging technology has demonstrated that resting-state neural activity-based functional connectivity across the brain network reorganizes under chronic pain in comparison with healthy states^1–4^. The resting-state neural activities revealed by functional neuroimaging, such as fMRI, can be used to objectively assess chronic pain in humans and animals^5,6^. Spontaneous activity, referred to as resting-state activity, exhibits spatiotemporally organized neuronal patterns in the absence of sensory inputs, even under general anesthesia^7^. The spatial representation of spontaneous neural activity in the cerebral cortex often corresponds closely to sensory-evoked responses^8,9^. Furthermore, repetitive cortical activation by excessive sensory stimulation increases the frequency of reactivation of cortical spontaneous activities that are similar to those evoked by sensory stimulation^10^. Therefore, the organized pattern evoked by a sensory experience is likely to shape spontaneous representations^11^. This hypothesis is possibly applicable to nociception: acute repetitive nociceptive inputs trigger an immediate reorganization of spontaneous cortical activities.

The insular cortex (IC) is one of the critical cortical foci for nociception^12,13^, and its spontaneous activity has been reported to change with chronic pain aggravating^14^. However, it is an open issue how nociception influences on dynamics of spontaneous neural activity in IC. This question is critical for understanding the characteristic mechanisms of pain because, dissimilar to other primary sensory cortices, IC receives not only multiple sensory signals, including gustatory, nociceptive, and visceral sensations, but also dense limbic inputs from the amygdala and hypothalamus^15,16^. Most of these connections are reciprocal, and thus, the information received in IC could be recurrent among the vast cortical network^17^.

Here, we tested the hypothesis that the organized pattern evoked by nociceptive stimuli forms a spontaneous representation in IC. Two approaches, wide-field calcium imaging and two-photon imaging, were applied to visualize the cortical activities of anesthetized GCaMP6s transgenic mice^18^. We compared the spontaneous neural activities before and after electrical stimulation of the periodontal ligament (PDL). Two different imaging techniques allowed us to identify the expected reactivation of nociception in spontaneous activity in the cortical area with single-cell resolution.

## Results

### Wide-field calcium imaging to visualize cortical responses to PDL stimulation

**Figure 1:**
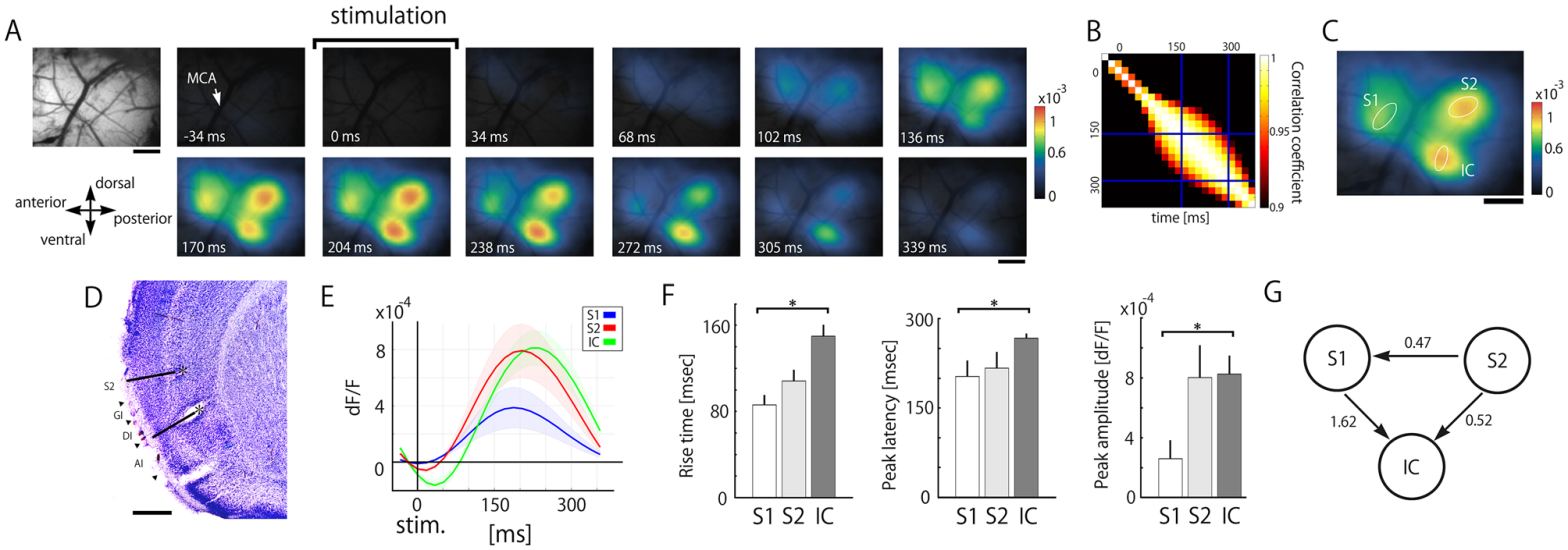
Identification of cortical areas responding to electrical stimulation of the PDL. (**A**) Representative cortical vascular pattern and color-coded cortical activities responding to PDL stimulation (0 ms) revealed by calcium imaging. Responses were obtained by averaging 435 trials. (**B**) A correlation matrix calculated from all pairs of frames. Areas with correlation coefficients (CC) over 0.9 are surrounded by a rectangle, which is defined as the stable response period. (**C**) PDL stimulation-evoked cortical activities obtained by averaging all frames during the stable period. Three cortical areas responsible for PDL stimulation are delineated by ellipses. The centers and major and minor axes for each ellipse are determined by principal component analysis of the local maximum of responses. (**D**) Nissl-stained coronal section. Asterisks indicate the locations of electric lesions applied to the edges of the region of interest. Black lines indicate the directions of the inserted electrodes. S2, granular IC (GI), and dysgranular IC (DI) are located between the two lesions. AI: agranular IC. (**E**) Averaged time courses in S1, S2, and IC. PDL stimulation was applied at 0 ms. (**A**)–(**C**) and (**E**) are obtained from the same mouse. (**F**) Kinetics of evoked responses, i.e., the rise time (left), latency to the peak (middle), and peak amplitude (right), are compared among S1, S2, and IC. *p < 0.05 (Steel–Dwass test). (**G**) Nonparametric multiplicative regression (NPMR)-based causality estimation of signal time courses across S1, S2, and IC. The arrows indicate a significant direction of causality. A larger value of C_NPMR_ means a stronger causal relationship. Information from S1 and S2 converged into IC. Scale bars in (**A**), (**C**), and (**D**) indicate 500 µm.

To identify the cortical foci to process nociceptive inputs delivered by electrical stimulation of the PDL, we performed wide-field calcium imaging of anesthetized GCaMP6s mice. In this example (Fig. 1A), the first cortical response was observed in the rostrodorsal part in reference to the cross point of the MCA and the rhinal vein 34 ms after PDL stimulation. Then, the second (68 ms after stimulation) and third responses (102 ms after stimulation) were evoked in the caudodorsal and caudoventral regions, respectively. Three responses reached a peak at approximately 170–204 ms and then sequentially disappeared in the order of emergence (Fig. 1A).

To quantify the responses to PDL stimulation in the cortex, we first performed correlation analysis across all frames. We found that the evoked spatiotemporal patterns of 152–271 ms constantly exhibited a high CC (> 0.9; Fig. 1B), indicating that the evoked patterns were almost identical. Therefore, we defined the period of 152–271 ms as the stable period. Next, we delineated the areal peak responses to PDL stimulation during the period by ellipses (Fig. 1C). Nissl staining of the imaged cortical areas confirmed that the location of the ellipses was cytoarchitecturally identified as the primary and secondary somatosensory cortices (S1, S2) and IC (Fig. 1D).

Figure 1E shows the signal time courses of the averaged signals from the ellipses in S1, S2, and IC. The rise time of IC was significantly longer than that of S1 (N = 6, *p* = 0.012; Fig. 1F, left). The peak latency was also slower in IC than in S1 (N = 6, *p* = 0.045; Fig. 1F, middle). The peak amplitude was also significantly higher in IC than in S1 (N = 6, *p* = 0.043; Fig. 1F, right).

To clarify the information flow among the three nociception-related areas, we conducted causality estimation using NPMR (see the Methods). In the framework of the NPMR-based causality estimation, we found three significant directions of information flow (S2** → **S1, S1** → **IC, S2** → **IC; Fig. 1G), indicating that the information from S1 and S2 converged into IC. The detected causality, in which cortical activities evoked by PDL stimulation were finally converged into IC^19^, is partially consistent with both the parallel and hierarchical processings^20–22^.

### PDL stimulation evolves neural reorganization in spontaneous IC activities

**Figure 2:**
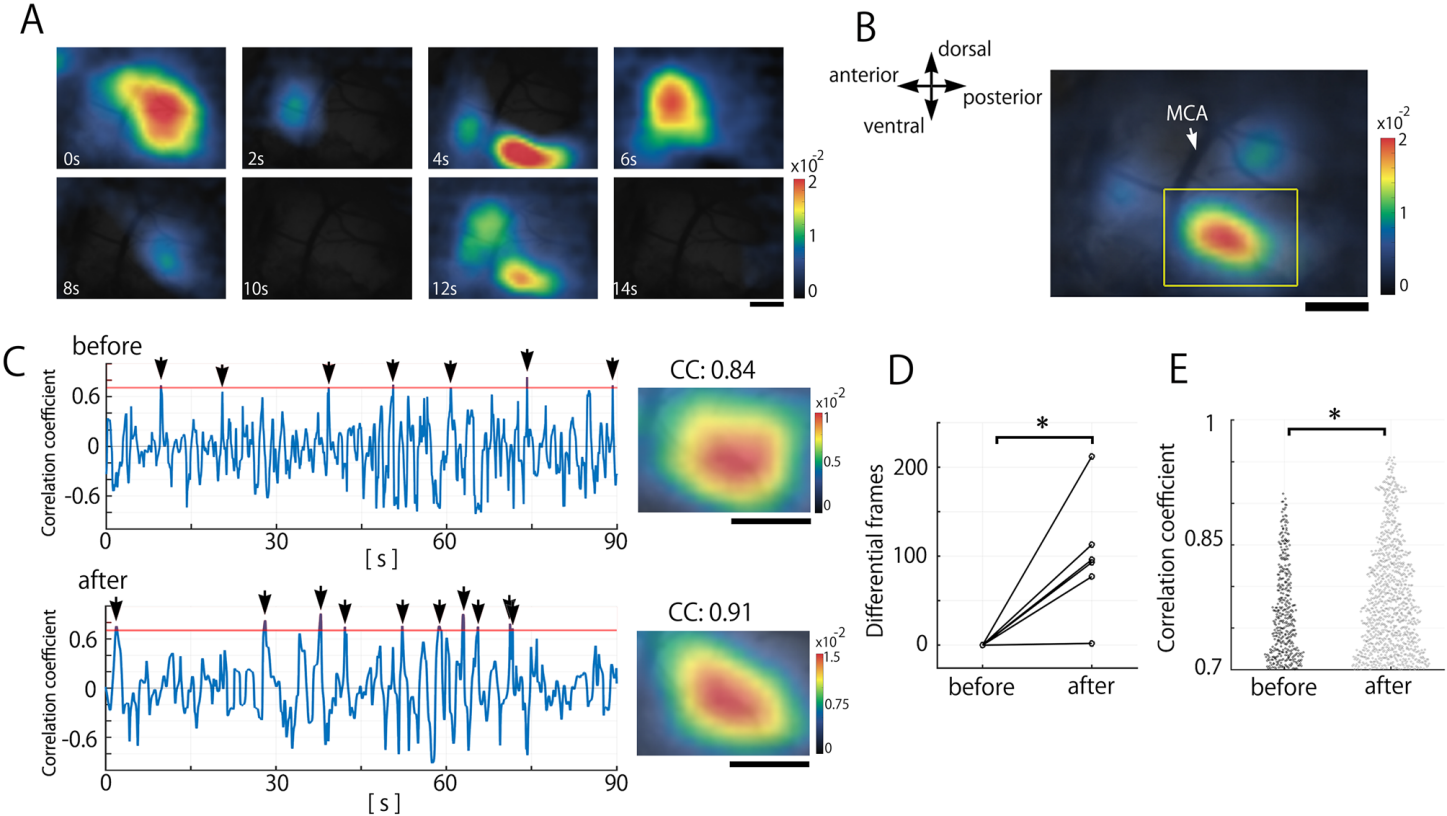
Effects of repeated electrical stimulation of the PDL on spontaneously emerging cortical activity. (**A**) Examples of spontaneous activity in the session before PDL stimulation, showing various activation patterns. (**B**) An example of the template image evoked by PDL stimulation. For spatial correlation analysis, the rectangular area was set to detect IC activity. (**C**) Time courses of spatial correlation coefficients (CC) between the template and each spontaneous frame in the session before (upper) and after (bottom) PDL stimulation. The threshold is set at the mean CC + 2 SD determined by the imaging session before the PDL stimulation (red horizontal lines). Arrowheads indicate the events over the threshold. Spontaneous patterns with maximum CC over the threshold are shown in the right column. Spontaneous activities over the threshold are observed in the sessions before and after PDL stimulation. (**D**) The number of frames that exhibited a CC larger than the threshold in the session after PDL stimulation was greater than that in the session before PDL stimulation (Wilcoxon signed-rank test; *p = 0.031). (**E**) Comparison of the CC of the frames that exhibited a larger CC than the threshold. PDL stimulation significantly augmented the CC (Mann–Whitney U test; ***p < 0.001). Scale bars in (**A**)–(**C**) indicate 500 µm.

Spontaneous IC activities before PDL stimulation emerged with various spatial patterns, and some spontaneous activities exhibited a similar pattern to the PDL stimulation-evoked response (Fig. 2A), as reported in other cortical areas^8,9^. To verify the hypothesis that responses to nociceptive inputs change and reorganize into spontaneous activity, we focused on spontaneous activities in IC, to which PDL stimulation-evoked signals finally reached, before and after repetitive PDL stimulation.

To quantify the frequency of the emergence of the PDL stimulation-evoked-like patterns in spontaneous activity, an evoked pattern template for correlation analysis was made by averaging the cortical responses evoked by PDL stimulation (Fig. 2B). This template was used to detect spontaneous activities matching the evoked pattern in the session before PDL stimulation. The spatial correlation analysis confirmed the emergence of patterns with a high correlation to the template (Fig. 2C, upper left).

Next, we conducted the same analysis on spontaneous activity after the session of PDL stimulation (2.5 Hz for 59 s). Consistent with the session before PDL stimulation, some spontaneous activity resembled the template, and the number of matched activities increased (Fig. 2C, bottom). To access the effect of nociceptive stimulation on spontaneous activities, we compared the number of matched images before and after the session of PDL stimulation: the emergence of matched images significantly increased (N = 6, *p* = 0.031; Fig. 2D). Furthermore, PDL stimulation significantly increased the CCs of spontaneous activities exhibiting patterns more similar to the evoked patterns (n_before_ = 413, n_after_ = 1006, *p* < 0.001; Fig. 2E), suggesting that repetitive nociceptive inputs reorganize the subsequent spontaneous activities in IC.

### Relationship of spontaneous activities between macroscopic and single neuron levels

**Figure 3:**
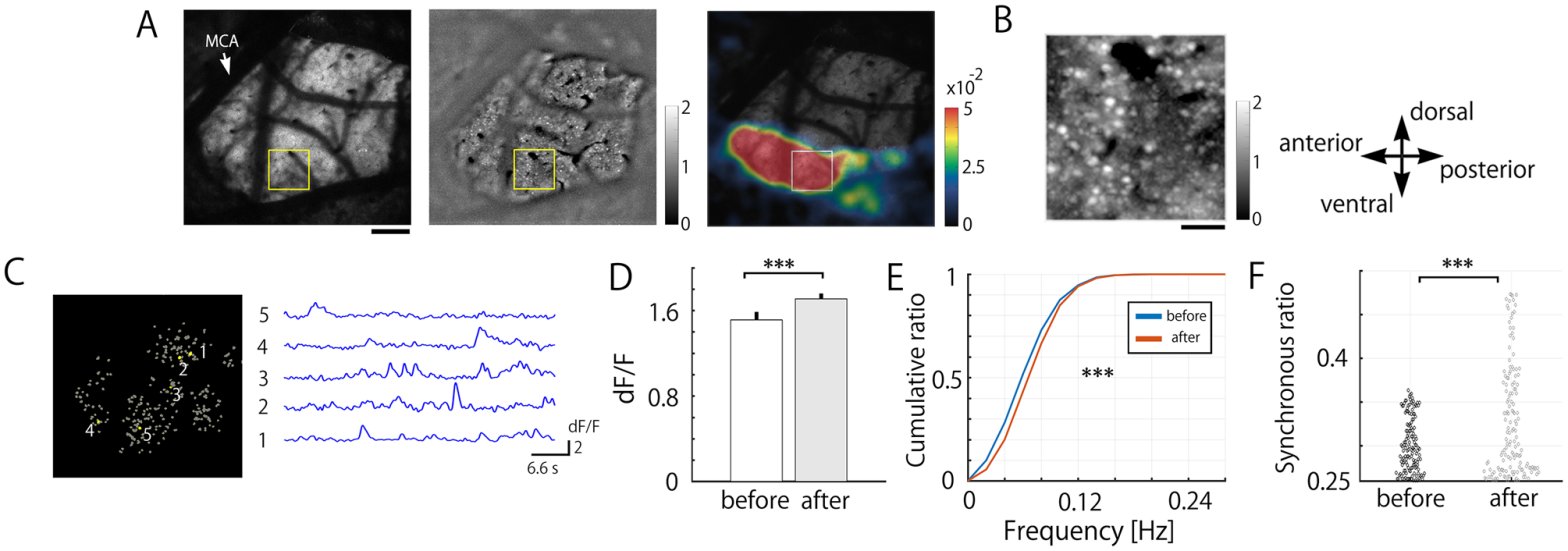
Two-photon calcium imaging of IC spontaneous neuronal activities in the session before and after PDL stimulation. (**A**) Detection of the IC region responding to PDL stimulation. *Left*, a vascular pattern of the cortical surface imaged by two-photon microscopy. Scale bar 200 µm. *Middle*, The time-stack average of spontaneous calcium activities in the same field as on the left. Each small bright dot indicates GCaMP6s-expressing neurons. *Right*, the merged image of the vascular pattern (left) and wide-field calcium imaging responding to PDL stimulation. (**B**) The enlarged image of the time-stack spontaneous calcium image shown in (**A**, *middle*). Scale bar 50 µm. (**C**) Masks of the regions of interest identified by the constrained nonnegative matrix factorization algorithm (*left*) and 5 representative spontaneous calcium signals (*right*) highlighted by yellow on the left. (**D**) The amplitude of spontaneous calcium signals in the session after PDL stimulation was larger than that before PDL stimulation (Mann–Whitney U test; ***p < 0.001). (**E**) A cumulative plot of the frequency of spontaneous calcium activities in the session before and after PDL stimulation (Kolmogorov–Smirnov test; ***p < 0.001). (**F**) PDL stimulation augmented spontaneous synchronous calcium activities across the neuronal population (Mann–Whitney U test; ***p < 0.001).

To understand the mechanism of the increase in macroscopic spontaneous IC activities, we recorded spontaneous calcium activities of each GCaMP6s-expressing layer II/III neuron (300 μm depth from the cortical surface) within the same field of wide-field imaging (Fig. 3A,B). Neurons fulfilling the criterion for statistical analysis were used on the subsequent quantitative analysis (see Method; Fig. 3C).

After the session of PDL stimulation, the amplitude (*dF/F*) and frequency of spontaneous calcium activities in each neuron significantly increased (n_before_ = 5982, n_after_ = 5703, *p* < 0.001; Fig. 3D, *p* < 0.001; Fig. 3E). The synchronous rate was also significantly augmented (n_before_ = 117, n_after_ = 133, *p* < 0.001; Fig. 3F).

These results indicate that repetitive PDL stimulation increases the synchronous activity of IC neurons responding to PDL stimulation, likely to contribute to the augmented macroscopic emergence of sensory-evoked-like spontaneous activity.

### Increased spontaneous activity is not attributed to ongoing pain

The increased spontaneous activity, which resembles PDL stimulation-evoked responses, is possibly induced by ongoing nociceptive inputs from an inflammatory reaction in the damaged PDL^23^ rather than by cortical plasticity. To examine this possibility, we compared spontaneously emerging activities with stereotypic evoked responses to PDL stimulation at the temporal profile of cortical activation patterns.

**Figure 4:**
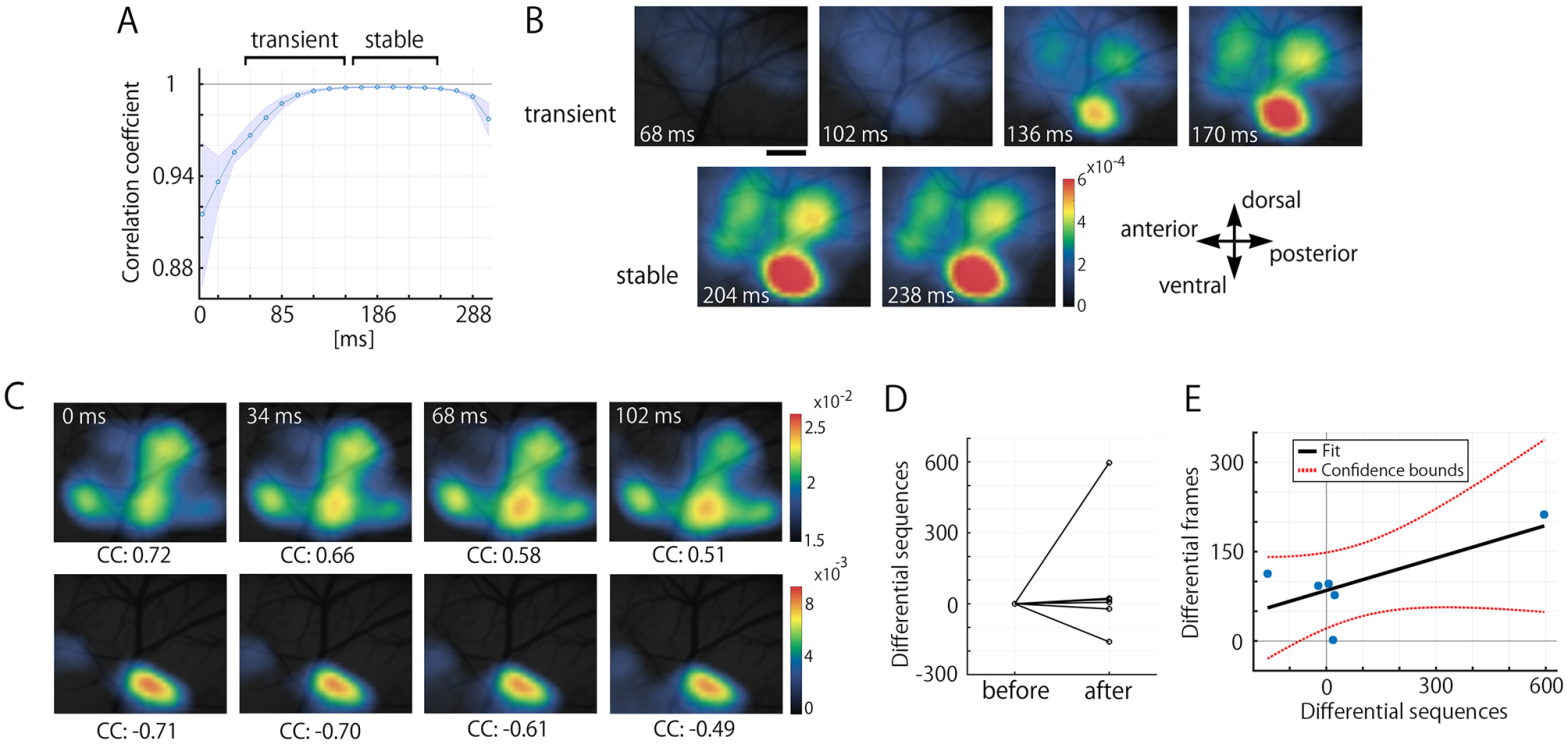
Detection of spontaneous cortical activities with a similar spatiotemporal pattern evoked by PDL stimulation. (**A**) A time course of three averaged consecutive spatial CCs. The SEM of the CC gradually decreased immediately after PDL stimulation (the transient period) and then reached the stable period. (**B**) A representative spatiotemporal sequential pattern responding to PDL stimulation in the transient (upper) and stable (bottom) periods. While the patterns gradually changed during the transient period, the stable period showed almost consistent spatial patterns. The scale bar indicates 500 µm. (**C**) Spontaneous sequential activities with a high CC (upper), i.e., high similarity to the activities evoked by PDL stimulation, and with a low CC (bottom), i.e., spontaneous IC activation without S1/S2 activities. (**D**) A comparison of the number of sequences with high similarity to the activities evoked by PDL stimulation, whose spatial correlation was over mean + 2 SD for prestimulus activities from 51 to 152 ms (the transient period), between the session before and after PDL stimulation. No significant difference was detected (Wilcoxon signed-rank test; *p* = 0.69). (**E**) Relationship between the spontaneously emerging IC activity (Differential frames) and evoked-like sequence (Differential sequences) in basis of the change of reactivation frequency after PDL stimulation. The overall regression was not statistically significant (F(1, 4) = [3.99], *p* = 0.12), suggesting no relationship between them.

In PDL stimulation-evoked responses, the averaged CC among three consecutive images at each time point after stimulus onset exhibited transient and stable periods (Fig. 4A). In the stable period, CC reached a plateau with a small SEM, whose features were visually characterized by stable and highly activated patterns (Fig. 4A,B, bottom). In contrast, the transient period (from 51 to 152 ms) exhibited a gradual increase in CCs with a relatively large SEM (Fig. 4A), visually exhibiting a gradual increase in the calcium signal with the activation sequence of S1** → **S2** → **IC (Fig. 4B, upper).

If the increased spontaneous activity is attributed to ongoing nociceptive inputs, the typical temporal profile of S1** → **S2** → **IC activation sequence in the transient period should be observed in the spontaneous activity. A representative of the spontaneously emerging evoked-like sequence resembling the patterns observed in the transition period is shown in Fig. 4C (upper). An example of spontaneously emerging IC activity without any correlation to evoked sequences by PDL stimulation is also shown in Fig. 4C (bottom). We examined the number of the sequence, i.e., the number of activation sequences of S1** → **S2** → **IC, among all spontaneous activities. There was no significant difference in the number of sequences between the sessions before and after PDL stimulation (N = 6, *p* = 0.69; Fig. 4D).

Next, we assessed a relationship between the spontaneously emerging IC activity and evoked-like sequence in basis of the change of reactivation frequency after PDL stimulation. In the case that the relationship between differential sequences and frames is positively correlated, the increased reactivation frequency of the sensory-evoked-like spontaneous patterns in IC could be due to ongoing nociceptive inputs^23^ rather than cortical plasticity. A linear regression revealed no significant relationship (F(1, 4) = [3.99], *p* = 0.12; Fig. 4E).

These results indicate that the increased spontaneous activity with spatiotemporal similarity to PDL stimulation-induced IC responses is likely due to cortical plasticity rather than ongoing nociceptive inputs whose influence on the frequency of typical activation sequences during spontaneous activity were not detected.

## Discussion

In this study, we tested the hypothesis that the organized pattern evoked by nociceptive stimuli forms a spontaneous representation in IC. First, we identified that nociceptive information flows evoked by PDL stimulation converged into IC. Next, in IC, we found that spontaneous stimulation-evoked-like activity tended to be increased after PDL stimulation. Finally, we ruled out the possibility that the altered spontaneous activity was attributed to ongoing nociceptive inputs by an inflammatory reaction in the damaged PDL. These finding suggest that the acute nociception-induced plasticity reorganizes spontaneous activity in IC under anesthesia.

Human neuroimaging studies have revealed that pain involves a set of brain regions referred to as pain-related cerebral substrates^24,25^. Due to the spatiotemporal limitations of the imaging studies, accurate activated regions and/or temporal profiles of the nociceptive processing often remain ambiguous, and therefore, two possible processings of nociception have been proposed: parallel processing^26^ and hierarchical processing^27^. In the present study using wide-field calcium imaging having high spatial and moderate temporal resolution, we detected three parcellated cortical areas with unique temporal profiles (Fig. 1E) and two significant directions of information flow by C_NPMR_ causality estimation (S1** → **IC, S2** → **IC; Fig. 1G) in response to PDL stimulation. These results suggest that nociceptive inputs to lower cortical areas (S1 and S2) are parallel and the inputs to the higher area (IC) are hierarchical, as a compromise between the two. This perspective may be anatomically supported by considering direct connections to S1 and S2 from the ventroposteromedial thalamic nucleus and posterior thalamic nucleus^20^, and the conventional viewpoint of hierarchical information processing from lower to higher areas^22^.

In the present study, we focused on the signal onset but not the rise time to the peak and the entire kinetics of Ca^2+^ signals such as half duration and decay time. GCaMP6s could respond to spike initiation as quickly as GCaMP6f does at signal onset^28^, although the entire kinetics is much slower. We have to pay attention to use the slow reporters including GCaMP6s for the analysis of fast events. Because the analysis at the limit of the signal resolution is heavily influenced by noise, decreasing the noise level is critical to discriminate the event kinetics. In the present study, the PDL stimulation-triggered averaging method made it possible to detect the fast sequential activation (S1 → S2 → IC), which were consistent across all animals we used. If GCaMP6s cannot distinguish the sequential activation pattern at 10 ms order, the order of activation in S1, S2, and IC would be random in each trial. However, even in a single trial, the sequential pattern of S1 → S2 → IC activation was detectable (Fig. S1). In addition, the obtained results were supported by other researches investigating cortical responses to noxious inputs^26,29^ and/or anatomical connections^20,22^. These findings support the idea that the difference in activation onset among S1, S2, and IC is detected by GCaMP6s.

Since the stimulation method applied in this study, electrical stimulation using bipolar electrodes, cannot selectively activate nociceptive fibers, there is a possibility that sequential cortical responses may be induced by the mixture of nociceptive and non-nociceptive stimuli. The magnetoencephalography studies conducted in noxious and innocuous stimulation demonstrated that the activation foci and their time courses were almost identical except for the onset delay in noxious stimulation, which could be attributed to the difference of peripheral and spinal conduction velocities between Aβ and Aδ fibers^26,29^. Based on these studies, the most-delayed IC activation induced by PDL stimulation may be induced by nociception. The selective stimulation to nociceptive and non-nociceptive fibers should be performed in future studies to dissect each contribution to the augmented spontaneous activity in the cerebral cortex.

Notably, there are several spatiotemporal inconsistencies between the present study and our line of extensive studies of nociceptive information processing in rat using a voltage-sensitive dye (VSD): The activated region in S2/ insular oral regions (IOR) cannot be spatially separated^30,31^. The signals were almost simultaneously increased within 20 ms in both S1 and S2/IOR. One explanation for these inconsistencies is the nature of the recorded signals. The VSD signal reflects the membrane potential, while the calcium signal relates to suprathreshold activities including action potentials^32^. S1, S2, and IC have different network architectures^20^, which may affect the spatiotemporal difference in the summation of postsynaptic potentials. Therefore, the latencies to generate action potentials are possibly inconsistent among these areas despite the similar latencies of postsynaptic potentials. VSD imaging can visualize PDL-induced dendritic signals in layer II/III pyramidal neurons, which abundantly exist and horizontally extend into layer I. This anatomical finding is a possible reason why activities in S2 and IOR cannot be distinguished by VSD imaging.

We demonstrated that spontaneous neural activity with a similar spatial pattern to that induced by PDL stimulation increased in the session just after repetitive PDL stimulation. In addition, these increased spontaneous activities were accompanied by an increase in synchronized pyramidal neuron activities. This PDL stimulation-induced augmentation of spontaneous IC activity resembles those in rodent models of chronic pain, in which inflammation or neuropathy with ongoing nociceptive inputs from damaged tissues largely involve^33,34^. However, it should be noted that their model yields the augment, utilizing long-term changes in cortical plasticity induced by a long stimulation period (more than 1 week). Thus, our results induced by acute PDL stimulation are likely to reflect neuronal replay^35^ that have been reported in areas of the neocortex and hippocampus as reemergence of neuronal patterns representing previous experience^10,36,37^. Short-term modifications in synaptic plasticity or in the intrinsic membrane properties are proposed one of the cellular mechanisms for the reactivation^38^. Reorganized spontaneous activity in IC may be attributed to short-term synaptic changes induced by experienced nociception. The plastic change in inhibitory synapses^39^ may also help to explain the increase in spontaneous activities and the decrease in the number of spontaneously active neurons, which were irrelevant to PDL stimulation.

Importantly, reactivation of the spontaneous activity decays with time in the visual cortex, whereas persistence of the reactivation depends on experienced or stimulated period, indicating the duration of the effect is modifiable^10^. Repetitive PDL stimulation in the present study was temporary, and thus, the increased spontaneous IC activities are likely to decline soon. However, in general, distinct from usual sensory inputs such as visual stimulation, nociceptive inputs can often accompany tissue damages, especially under severe nociception. Ongoing nociceptive inputs from the damages may prolong the survival time of the neuronal replay.

Reactivation of the sensory-evoked activity has been hypothesized to strengthen the selective neuronal connections between neurons representing the experienced information^40^, working as memory consolidation^41^. Spontaneously emerging nociception-related activity may continue even after the recovery from tissue damages, and may enhance long-term synaptic changes. Possible causal factors are diseases accompanying inflammation and nerve injury in the orofacial area, a part of which induce persistent pathological pain even after the recovery from the peripheral abnormalities such as lesions^42,43^. Inflammation and nerve injury repetitively activate the trigeminal nerve, and as a result, these pathological conditions may increase the nociception-related spontaneous IC activities. Indeed, spontaneous activity in IC has been reported to change with chronic pain aggravating^14,44,45^. Thus, preventing spontaneous activity from representing nociceptive information probably contributes to a promising prophylaxis for chronic pain.

## Methods

The Animal Experimentation Committee of Nihon University approved animal housing, breeding, and experimental procedures (AP16D028, AP16D029, AP17D003, AP18DEN024, AP18DEN047, AP19DEN025). All studies were performed in strict accordance with the guidelines of NIH, and in compliance with the ARRIVE guidelines. Efforts were made to minimize animal suffering and to reduce the number of animals examined.

### Animals

Twelve *Thy1*-GCaMP6s transgenic mice^18^ of either sex obtained from Jackson Laboratories (C57BL/6J-Tg (*Thy1*-GCaMP6s) GP4.3Dkim/J; Jax stock #024275) were anesthetized with intraperitoneal injections of atropine methyl bromide (0.1 mg/kg) and urethane (1.2 g/kg) and then fixed to a stereotaxic frame. The body temperature of the mice was maintained at approximately 37 °C (BWT-100, Bio Research Center, Tokyo, Japan). After removing the scalp and skin between the left eye and ear under subcutaneous local anesthesia of ropivacaine (AstraZeneca, Osaka, Japan), resection of a part of the temporalis muscle and zygomatic arch (Fig. S2A,B), and craniotomy (approximately ~ 4 × 4 mm for wide-field imaging or ~ 2 mm diameter for two-photon imaging) were performed along squamosal and sphenosquamosal sutures (Fig. S2B,C) referring to the middle cerebral artery (MCA) and caudal rhinal vein as surgical landmarks for IC^46^. The approximate coordination for the center of imaged area was anteroposterior + 1.0 mm from Bregma^47^ and dorsoventral + 2 mm from squamosal suture. The dura matter was kept intact. The cranial window was filled with 2% agar and sealed with a glass coverslip. We did not detect the aberrant cortical activity reported in other lines of transgenic mice expressing GCaMP6 genetically encoded calcium sensors^48^.

### Electrical stimulation of PDL

Bipolar electrodes made from a formvar-insulated nichrome wire (diameter = 66 μm) were inserted into the right mesial maxillary first molar PDL^49^. Voltage pulses (5 ms, 5 V) were applied at 2.5 Hz for 59 s for wide-field imaging, and 10 trains of pulses (100 μs, 20 Hz, 5 V) were applied 400 times with a 9.8 s interval for two-photon imaging using a stimulator unit (STG2008, Multi Channel Systems, Reutlingen, Germany). Spontaneous activity in the session after PDL stimulation was recorded 5 min after the end of the stimulation. In our preparation, the intensity threshold of electrical PDL stimulation that induced jaw-opening reflex (JOR) was approximately 3.5 V. The 5 V-PDL stimulation in the present and our previous studies^19^ corresponds to the 1.4-fold intensity of the JOR threshold.

### Wide-field calcium imaging

A microscope (BX51WI, Olympus, Tokyo, Japan) equipped with a 1.5 × objective (10447050, Leica Microsystems, Wetzlar, Germany), a mirror unit (U-MNIBA2, Olympus) and a CMOS camera (MiCAM03, Brainvision, Tokyo Japan) was used to record wide-field calcium signals. The cortical surface was illuminated by the epifluorescence system with a 465 nm LED light source (LEX2-LZ4-B, Brainvision). Images with a size of 256 × 256 pixels and a pixel width of 19.5 μm were captured for 99 s and 59 s at a 59 Hz frame rate to obtain spontaneous and evoked activities, respectively, in 6 mice.

### Two-photon calcium imaging

The calcium signals of individual neurons were recorded from 6 mice using two-photon microscopy (FVMPE-RS, Olympus). The excitation wavelength was 940 nm, and imaging was conducted with a 10 × water-immersion objective (XLPLN10XSVMP, Olympus) placed 300 μm from the cortical surface. The emitted light was detected by a dichroic mirror (570 nm) with bandpass filters (495–540 nm and 575–645 nm; FV30-FGR, Olympus). Images (512 × 512 pixels, pixel width = 2.49 μm) were recorded for 99 s in the spontaneous session. The sampling rate was 30 Hz.

### Data analysis

Data analyses were conducted with ImageJ (NIH) and custom-written code in MATLAB (Mathworks, Natick, USA). For wide-field imaging, the original images were reduced to 85 × 85 pixels. The extracted calcium signals were corrected by eliminating the signal trend and heartbeat contamination^50^. The resultant signals were temporally smoothed in the 0.3–3.0 Hz band^51^. To improve SNR in the images, spatial smoothing was applied by a Gaussian kernel with a standard deviation of 59 μm. Evoked signals in the fluorescence intensity of each pixel relative to the initial intensity were calculated (*dF/F*) to correct the uneven indicator expression^52^. Spontaneous signals were obtained by dividing the signals by their average over time. To measure the mean evoked response, all recorded signals were averaged, and the normalized difference to the averaged baseline recorded 50 ms before stimulation was calculated. In the evaluation of spatial pattern similarity, we compared optical frames with a template image using the correlation coefficient (CC). Significant similarity was defined as a value exceeding the mean CC + 2 SD calculated from the imaging session before the PDL stimulation. To detect the evoked-like sequences, seven sequential frames (from 51 to 152 ms) during the transition period (see the last section on “Results” for the definition) of the averaged-evoked response were compared to those from spontaneous activity. If the minimum CC from seven frames was higher than the mean CC + 2 SD obtained from the imaging session before the PDL stimulation, the corresponding spontaneous sequence was regarded as an evoked-like sequence.

To evaluate single-cell calcium transitions recorded by two-photon imaging, frame alignment was first performed using TurboReg, a plugin of ImageJ. Regions of interest corresponding to the cell body in the corrected images were then automatically selected with the constrained nonnegative matrix factorization algorithm^53^, and the mean neuronal signal (Fn) was extracted for each region. We also estimated the local neuropil signal (Fnp) from a surrounding band of each cell, whose width was 3 μm and was placed 2 μm apart from the edge of the cell to reduce the possible overlap between the band and cell (excluding adjacent cells)^54^, and subtracted a fraction of it from the neuron signal F = Fn − 0.7 Fnp^55^. The change in fluorescence was represented as *dF/F*, where each time point of the calcium signal was subtracted from the mean signals over the recording and then divided by the same mean. Neurons whose calcium transients went over baseline + 2 SD were referred to as active and were used for statistical analysis. Because the synchronous activity indicates that a set of neurons are active at the same time, we defined the synchronization rate of neuronal activity as the ratio of neurons that were coactive in each frame relative to the total number of recorded neurons. Then, the synchronous threshold was set to a mean + 2 SD from the imaging session before the PDL stimulation.

### Nonparametric multiplicative regression-based causality estimation

Given two time series, X and Y, X is predicted by only its past information of X or by the past information and another variable (Y). If the prediction is better in the latter case, X and Y have a causal relationship^56^. Nonparametric multiplicative regression (NPMR) was used to evaluate the cause and effect between the two time series (for details^57^). Statistical significance was assessed by the surrogate method (α = 0.05;^58^). Positive values of causality estimation (C_NPMR_) imply that the addition of past information from other time series results in better prediction; therefore, there is a significant causal relationship between the time series, and vice versa.

### Histology

After the recording, electrolytic lesions were made by applying an 8.5 V, 5-second DC to the four corners of the imaged region. Animals were deeply anesthetized with isoflurane (5%), and decapitated. The heads were submerged into 4% paraformaldehyde solution for fixation, then cryoprotected in 30% sucrose in PBS until they sank. The brains were removed, frozen and coronally sectioned with a sliding microtome (Leica Biosystem, Nussloch, Germany) set at 50 μm. Nissl staining of the brain sections was performed by the conventional method^58^.

### Statistical analysis

N and n indicate the number of mice and the recorded frames or neurons, respectively. For multiple comparisons, the Steel–Dwass test was performed. If the samples were paired, the Wilcoxon signed-rank test was used; otherwise, the Mann–Whitney U test was adopted. To compare the distributions, the Kolmogorov–Smirnov test was conducted. For all statistical comparisons, *p* < 0.05 was considered to be significant.

## Supplementary Information

Supplementary Figures.

## Data Availability

The datasets generated during and/or analysed during the current study are available from the corresponding author on reasonable request.
