# Supplementary material for: Repetitive nociceptive stimulation increases spontaneous neural activation similar to nociception-induced activity in mouse insular cortex
Source: Sci Rep. 2022 Sep 7;12:15190. doi: 10.1038/s41598-022-19562-1 (PMC9452502; doi:10.1038/s41598-022-19562-1)
Supplement: Supplementary file 1 — Supplementary Figures. [file 41598_2022_19562_MOESM1_ESM.pdf]

Repetitive nociceptive stimulation increases spontaneous neural activation similar to nociception-induced activity in mouse insular cortex

Shutaro Kobayashi [1,2†], Kazunori O'Hashi [1,3,4,†,*], Masayuki Kobayashi [1,3,5,*]

[1] *Department of Pharmacology, Nihon University School of Dentistry, 1-8-13 Kanda-Surugadai, Chiyoda-ku, Tokyo 101-8310, Japan*

[2] *Department of Oral Surgery, Nihon University School of Dentistry, 1-8-13 Kanda-Surugadai, Chiyoda-ku, Tokyo 101-8310, Japan*

[3] *Division of Oral and Craniomaxillofacial Research, Dental Research Center, Nihon University School of Dentistry, 1-8-13 Kanda-Surugadai, Chiyoda-ku, Tokyo 101-8310, Japan*

[4] *Department of Mental Disorder Research, National Institute of Neuroscience, National Center of Neurology and Psychiatry (NCNP), 4-1-1 Ogawa-Higashi, Kodaira, Tokyo 187-8502, Japan*

[5] *Molecular Imaging Research Center, RIKEN, 6-7-3 Minatojima-minamimachi, Chuo-ku, Kobe 650-0047, Japan*

[†] *These authors contributed equally to this work.*

[*] ***Corresponding authors***:

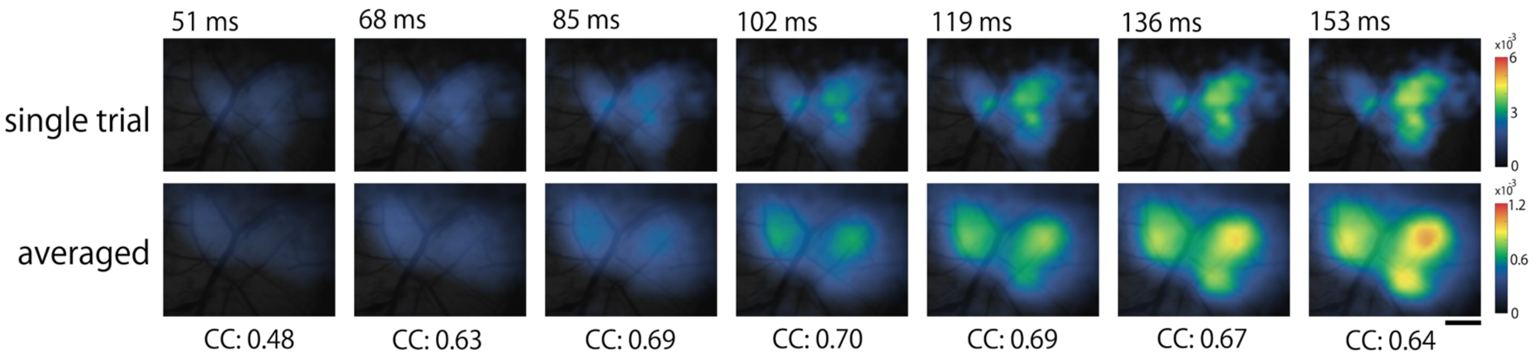

Figure S1. A representative comparison of the spatiotemporal sequential pattern evoked by PDL stimulation during the transient period between single and averaged trials. Scale bar: 500 µm.

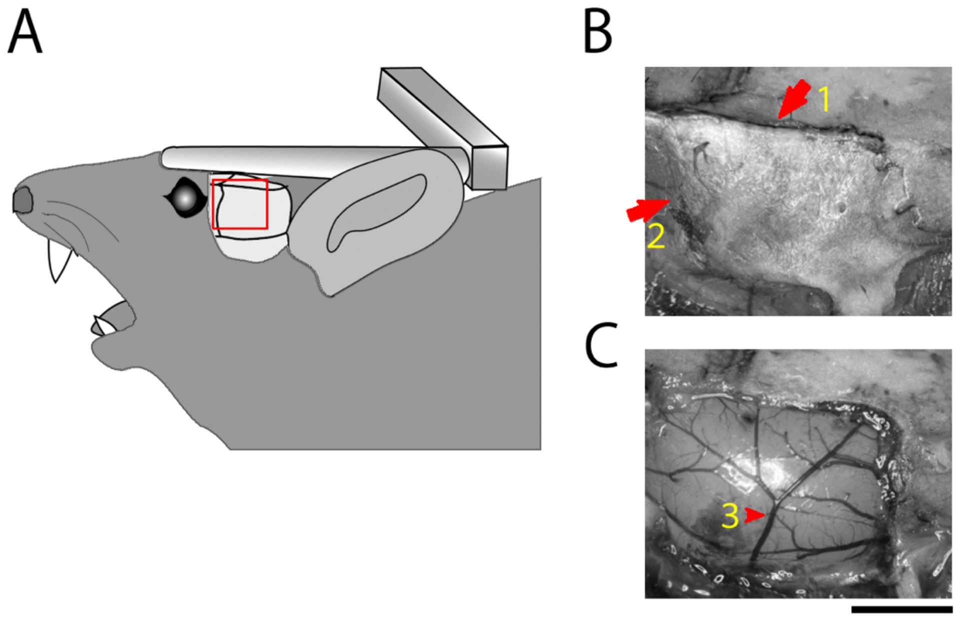

Figure S2. Preparation for a cranial window.
(A) A schematic lateral view for the surgical area. **(B)** An enlarged image of a temporal bone, which corresponds to the red rectangle shown in (**A**). Arrow 1 and 2 indicate squamosal and sphenosquamosal sutures, respectively. **(C)** An example of a cranial window. Arrowhead 3 points to the middle cerebral artery (MCA). Scale bar: 2 mm.
